# SC List-Flip Decoding of Polar Codes by Shifted Pruning: A General Approach

**DOI:** 10.3390/e24091210

**Published:** 2022-08-29

**Authors:** Mohammad Rowshan, Emanuele Viterbo

**Affiliations:** Department of Electrical and Computer Systems Engineering, Monash University, Melbourne, VIC 3800, Australia

**Keywords:** polar codes, successive cancellation, list decoding, tree pruning, shifted pruning, segmented decoding

## Abstract

In the successive cancellation (SC) list decoding, the tree pruning operation retains the *L* best paths with respect to a metric at every decoding step. However, the correct path might be among the *L* worst paths due to the imposed penalties. In this case, the correct path is pruned and the decoding process fails. shifted pruning (SP) scheme can recover the correct path by additional decoding attempts when decoding fails, in which the *pruning window* is shifted by κ≤L paths over certain bit positions. A special case of the shifted pruning scheme where κ=L is known as SCL-flip decoding, which was independently proposed in 2019. In this work, a new metric that performs better in particular for medium and long codes is proposed, and nested shift-pruning schemes are suggested for improving the average complexity.

## 1. Introduction

Polar codes proposed by Arıkan in [1] are the first class of constructive channel codes that were proven to achieve the symmetric (Shannon) capacity of a binary-input discrete memoryless channel (BI-DMC) using a low-complexity successive cancellation (SC) decoder. However, the error correction performance of polar codes under SC decoding is far from that of other popular codes, such as LDPC codes and Turbo codes. To address this issue, successive cancellation list (SCL) decoder was proposed in [2] which yields an error correction performance comparable to maximum-likelihood (ML) decoding at high SNR. Furthermore, it was shown that concatenation with cyclic redundancy check (CRC) bits [3] and convolutional codes [4,5] can improve the performance of polar codes.

When SC or SCL decoding fails, we may be able to correct the error(s) in additional decoding attempts. Bit-flipping [6] is a popular method to improve the error correction performance of the SC decoder b single or multiple flipping of the low-reliability bit(s) in each re-decoding attempt. The numerical experiments in [6] showed a predominant portion of the decoding failures occur by a single error in bit estimation due to channel noise. Thus, by finding the first erroneous bit and flipping the estimated value, error propagation can be prevented. This idea was further improved by using an improved metric for single and multiple flipping in [7], which was later computationally simplified [8]. The performance of these methods can approach the performance of SCL decoding with a moderate list size L=2, or in the case of employing multiple flipping, it can reach to the performance of L=4 or 8. However, in terms of complexity, nested/multiple bit-flipping may require a massive number of attempts (tens or hundreds of attempts), which makes multiple flipping impractical. Note that the receiving messages from the physical channel cannot wait for such a huge number of iterations, and we cannot afford to have a huge input buffer and output buffer to regulate the input and output bit streams.

The attempt to re-decode the failed SCL decoding was first proposed in [9] by gradually increasing the list size (*L*) by a factor of two until reaching the predefined maximum list size or succeeding in decoding. However, increasing the list size not only contributes to a larger computational time complexity but also requires enough hardware resources to support that. Additionally, as the results showed, the performance gain beyond list size L>32 is less than 0.1 dB for the increase in the list size by a factor of two, which is obtained at a very high cost of doubling the resources. Hence, this scheme is out of the scope of this work and makes the comparisons unfair. In [10], a bit-flipping scheme based on a modified critical set was employed in CRC-aided SCL decoding. Later, we proposed the shifted pruning scheme in [11] where the pruning window was shifted by κ=L (i.e., selecting the worst *L* paths in the sorted list instead of the best ones) at the critical bit coordinates. This work was submitted to ITW2019 on 10 April 2019, presented on August 26 in Visby, Sweden, and published on IEEE Xplore on 10 February 2020. Later, this scheme was improved in [12] by employing a computationally simple LLR-based metric and a variable κ (e.g., κ=L/2). The variable shift could correct the error by finding an approximate location of the elimination of the correct path. Moreover, the original shifted pruning scheme in [11] was improved in [13] by an ordered critical set in an adaptive list size scheme. Independently of us, similar work was proposed in [14]. In this work, inspired by [7], a probabilistic metric was employed that had a poor performance on medium and long codes. A different metric based on a probability ratio was suggested in [15]. The approximation of this metric in the log domain is similar to the path metric range (PMR) suggested in [12,16,17]. Nevertheless, this metric also suffers from poor performance for relatively long codes. Furthermore, the dynamic SCflip scheme for SC decoding in [7] was adapted to its SC list decoding counterpart in [18].

To improve the comparability of different bit positions in terms of the possibility of path elimination, we introduce a new metric in this work. Additionally, toward avoiding multiple eliminations of the correct path throughout the decoding, we extend this scheme by a low-complexity segment-wise multiple shifting. Note that in the generalized shifted pruning, (1) κ does not need to be *L*, but it can vary from 0 to *L*, and (2) we can perform the shifting multiple times in a nested fashion. The main contributions of this work are as follows:

- The metric for prioritizing the shifting positions is improved. This metric is computationally simple. Furthermore, it performs significantly better on medium and long codes, compared with the available metrics.
- Nested shifting is proposed for further improvement of the error correction performance. Due to the high complexity of multiple shifting, we limit the nesting scope to double shifting only. Moreover, we limit the double-shifting to a specific segment of the code. The shifted pruning based on ordered pairs improves the FER performance at a cost of a slight increase in complexity.

## 2. Preliminaries

A polar code of length $N=2^{n}$ with *K* information bits is constructed by choosing *K* good bit-channels in the polarized vector channel for transmitting the information bits and optional auxiliary CRC or parity bits. The indices of these bit-channels are collected in the set A. The rest of the N−K bit-channels are used to transmit known values, zero by default. For more details on the construction of polar codes and the polarization phenomenon, see [1]. The polar codes’ generator matrix is $G_{N}=G_{2}^{⊗n}$, where $G_{2}\overset{\Delta }{=}\left[\begin{array}{cc} 1 & 0\\ 1 & 1 \end{array}\right]$, and ${\left(\cdot \right)}^{⊗n}$ denotes the *n*-th Kronecker power [1]. Polar codes are encoded by $x_{0}^{N-1}\;=\;u_{0}^{N-1}G_{N}$ where $u_{0}^{N-1}=\left(u_{0},u_{1},\ldots ,u_{N-1}\right)$ is the input vector, including frozen and non-frozen bits, and $x_{0}^{N-1}\;=\;\left(x_{0},x_{1},\ldots ,x_{N-1}\right)$ represents the coded bits vector. Let $y_{0}^{N-1}\;=\;\left(y_{0},y_{1},\ldots ,y_{N-1}\right)$ denote the output vector of a noisy channel.

Let $\lambda_{j}^{i}$ denote the log-likelihood ratio (LLR) of bit *i* at stage *j* of the factor graph of SC decoding. The non-frozen bits are estimated successively based on the decision LLR, $\lambda_{0}^{i}$, via a one-time pass through the factor graph.

In SC decoding, the final decision on non-frozen bit values, i.e., ${\hat{u}}_{i}$ where i∈A, is made by the maximum likelihood (ML) rule $h(\lambda_{0}^{i})$ in (1), which depends on the estimation of previous bits, i.e., ${\hat{u}}_{0},\ldots ,{\hat{u}}_{i-1}$.

(1) $${\hat{u}}_{i}=h\left(\lambda_{0}^{i}\right)=\{\begin{array}{cc} 0 & \lambda_{0}^{i}=\ln\frac{P(Y,{\hat{u}}_{0}^{i-1}|{\hat{u}}_{i}=0)}{P(Y,{\hat{u}}_{0}^{i-1}|{\hat{u}}_{i}=1)}>0,\\ 1 & \text{otherwise} \end{array}$$

However, the SCL decoding considers both possible values, $u_{i}=0$ and $u_{i}=1$, and finds a path through the binary tree, which has the highest probability to be the transmitted sequence $u_{0}^{N-1}$. For optimal decoding, the probability to be *maximized* is

(2) $$\begin{array}{c} P\left({\hat{u}}_{0}^{N-1}|y_{0}^{N-1}\right)=\prod_{t=0}^{N-1}P\left({\hat{u}}_{t}|{\hat{u}}_{0}^{t-1},y_{0}^{N-1}\right) \end{array}$$

However, neither in SC decoding nor in SCL decoding can we traverse the entire decoding tree, i.e., considering all the paths. Therefore, the solution obtained from the decoding can be sub-optimal. In SCL decoding, the probability of partial path *l* representing the sequence ${\hat{u}}_{0}^{i-1}=\left({\hat{u}}_{0},{\hat{u}}_{1},\ldots ,{\hat{u}}_{i-1}\right)$ is computed by

(3) $$P\left({\hat{u}}_{0}^{i}\left[l\right]|y_{0}^{N-1}\right)=\prod_{t=0}^{i}P\left({\hat{u}}_{t}\left[l\right]|{\hat{u}}_{0}^{t-1}\left[l\right],y_{0}^{N-1}\right)$$

In practice, it is more convenient and practical to deal with the logarithm of (2). Hence, we take the logarithm of (3). Since log(x)<0 for x<1, we multiply the resulting logarithm by −1 to have a positive metric. Therefore, we obtain the following logarithmic *path metric*:

(4) $$\begin{array}{c} PM_{l}^{\left(i-1\right)}=-\log P\left({\hat{u}}_{0}^{i-1}\left[l\right]|y_{0}^{N-1}\right)\\ \;=-\sum_{j=0}^{i-1}\log P\left(\hat{u_{j}}\left[l\right]|{\hat{u}}_{0}^{j-1}\left[l\right],y_{0}^{N-1}\right) \end{array}$$

for the sequence ${\hat{u}}_{0}^{i-1}$ at position *l*.

Now let the sequence ${\hat{u}}_{0}^{i}$ be obtained by appending ${\hat{u}}_{i}$ to ${\hat{u}}_{0}^{i-1}$ on path *l* and suppose ${\hat{u}}_{i}$. The path metric for this longer sequence is

(5) $$PM_{l}^{\left(i\right)}=-\sum_{j=0}^{i}\log P\left({\hat{u}}_{j}\left[l\right]|{\hat{u}}_{0}^{j-1}\left[l\right],y_{0}^{N-1}\right)=PM_{l}^{\left(i-1\right)}+\mu_{l}^{\left(i\right)}$$

where $\mu_{l}^{\left(i\right)}=-\log P\left({\hat{u}}_{i}\left[l\right]|{\hat{u}}_{0}^{i-1}\left[l\right],y_{0}^{N-1}\right)$ denotes the *branch metric*, and $PM_{i}^{\left(-1\right)}=0$. Hence, the path metric along a path ending at bit *i* is obtained by adding the path metric ending at bit i−1 to the branch metric at bit *i*. Throughout this paper, we assume that the index *l* indicates the position of a sorted path list, i.e., $PM_{1}^{\left(i\right)}\leq PM_{2}^{\left(i\right)}\leq \cdots \leq PM_{L}^{\left(i\right)}$ and $l_{c}$ denotes the index of the correct path.

To simplify the arithmetic operation, we can define $\mu_{i}$ in (5) as

(6) $$\begin{array}{c} \mu_{l}^{i}=-\log P\left({\hat{u}}_{i}\left[l\right]|{\hat{u}}_{0}^{i-1}\left[l\right],y_{0}^{N-1}\right)\\ \;=-\log\left(\frac{e^{\left(1-{\hat{u}}_{i}\left[l\right]\right)\lambda_{0}^{i}\left[l\right]}}{e^{\lambda_{0}^{i}\left[l\right]}+1}\right)=\log\left(1+e^{-\left(1-2{\hat{u}}_{i}\left[l\right]\right)\lambda_{0}^{i}\left[l\right]}\right) \end{array}$$

where the last equality holds only for ${\hat{u}}_{i}\left[l\right]=$ 0 and 1. For the value of ${\hat{u}}_{i}\left[l\right]$ that equals $h(\lambda_{0}^{i}\left[l\right])$, the term $e^{-\left(1-2{\hat{u}}_{i}\right)\lambda_{0}^{i}}=e^{-|\lambda_{0}^{i}|}$ is small and hence $\log(1+\;e^{-|\lambda_{0}^{i}|})\approx 0$. Otherwise, we can approximate $\log\left(1+e^{|\lambda_{0}^{i}|}\right)\approx \left|\lambda_{0}^{i}\right|$ for $|\lambda_{0}^{i}|>1$. Thus

(7) $$\mu_{l}^{i}=\mu_{l}^{i}\left(\lambda_{0}^{i}\left[l\right],{\hat{u}}_{i}\left[l\right]\right)\;\approx \;\{\begin{array}{cc} 0 & \mathrm{if}\;{\hat{u}}_{i}\left[l\right]=h\left(\lambda_{0}^{i}\left[l\right]\right)\\ |\lambda_{0}^{i}\left[l\right]| & \mathrm{otherwise} \end{array}$$

As (7) shows, the path of the less likely bit value is penalized by $|\lambda_{0}^{i}\left[l\right]|$. At each decoding step, the *L* paths with the smallest metrics are chosen among 2L paths and stored in ascending order from $PM_{1}^{\left(i\right)}$ to $PM_{L}^{\left(i\right)}$. After decoding the last bit, the path with the smallest path metric, i.e., $PM_{1}^{\left(N-1\right)}$, or the path that passes the CRC, is selected as the estimated sequence.

Additionally, when the SCL decoding fails, the correct path might still be in the list but not in the position of the most likely path. Adding an *r*-bit CRC as an outer code to the information bits can assist the decoder in error detection and finding the correct path among the *L* paths. However, this concatenation increases the polar code rate to (K+r)/N. In this paper, P(N,K+r) denotes a polar code of length N with K information bits concatenated with *r*-bit CRC.

The correct path is pruned from the list when it has a relatively large PM due to the accumulated penalties and falls among the paths with indices L+1 to 2L in the sorted list. Thus, one can think of changing the rule for tree pruning to avoid the elimination of the correct path.

**Figure 1:**
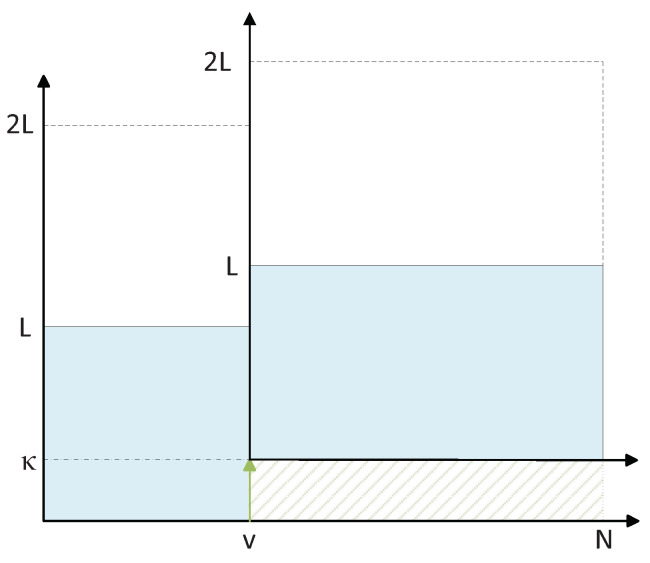
Shifting the pruning window by κ paths during list pruning operation at bit v∈V. Note that at coordinate *v*, the whole coordinate system is shifted up.

When the decoding fails in the first attempt in the list decoding of polar codes, an effective way to recover the correct path is to retain the *L* paths with the largest PMs (instead of *L* paths with the smallest PM values) over a bit (or bits in multiple decoding attempts) where there is a high probability of elimination of the correct path. This operation which is shown in Figure 1 is named shifted pruning [11] because in the process of selection of the paths to remain in the list, the reference for the first path in the *pruning window* is shifted by κ=L, i.e., we select the path κ+1 to path L+κ (instead of path 1 to path *L*) in the PM-based sorted list of paths.

**Figure 2:**
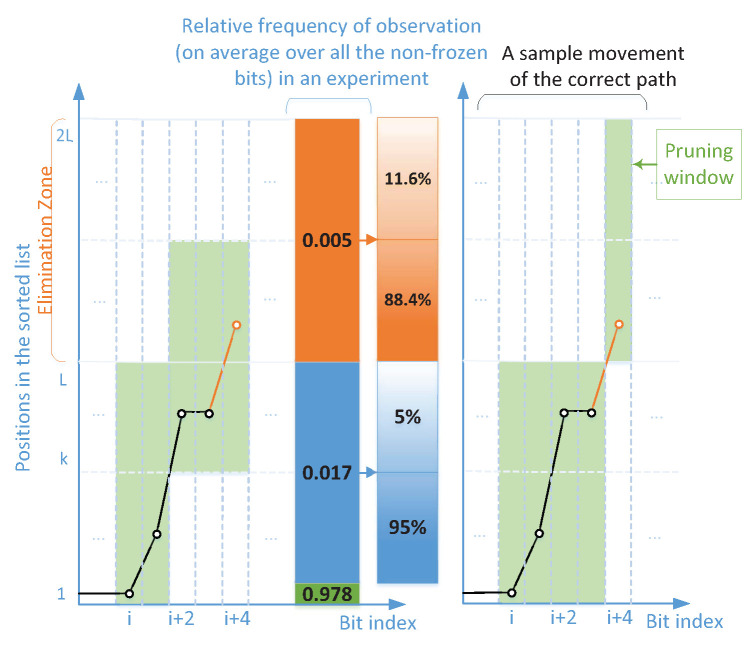
A sketch of L/2-shift (κ=L/2) vs. *L*-shift (κ=L) during pruning operation.

Instead of the accurate identification of the bit index, where the correct path is pruned, it was shown in [12] that when κ<L, e.g., κ=L/2, depending on the position of the correct path in the sorted list, it can still avoid the elimination of the correct path, even when the exact elimination index is not among the top *T* candidate positions for shifting. Figure 2 illustrates an example of elimination due to three penalties at bit i+1, i+2, and i+4, recovered by κ=L (right) and κ=L/2 (left). In general, κ can be different at different bit positions.

## 3. An Effective Metric

The metrics proposed in [12,14,15] do not provide a good FER performance with medium and long codes. The metric in [14] computes the probability of finding the correct path among paths l=L+1,...,2L at each non-frozen bit position *i* by summing the probability of each of these paths to be the correct path, i.e., $M\left(i\right)=\sum_{l=L+1}^{2L}P\left({\hat{u}}_{0}^{i}\left[l\right]|y_{0}^{N-1}\right)$ where

(8) $$P\left({\hat{u}}_{0}^{i}\left[l\right]|y_{0}^{N-1}\right)=P\left({\hat{u}}_{0}^{i-1}\left[l\right]|y_{0}^{N-1}\right)P\left({\hat{u}}_{i}\left[l\right]|{\hat{u}}_{0}^{i-1}\left[l\right],y_{0}^{N-1}\right)$$

Obviously, as *i* increases, $P({\hat{u}}_{0}^{i}\left[l\right]|y_{0}^{N-1})$ becomes smaller due to multiplication in (8) and the fact that P(.)<1. Therefore, this metric by nature is biased toward small indices. That is, it gives higher weight to the low-reliability bits with smaller indices i∈A. As a result, we observe small indices more than the larger ones among the top *T* positions after sorting the metric values of all non-frozen positions. This metric may perform well on short codes, such as P (256,128), but as the numerical results show in [14], the gain becomes small at longer codes, such as codes with length 1 k bits. The metrics suggested in [12] also underperform when it comes to longer codes.

We are interested in a metric for the list decoding that resembles the reliability of bit-channels in a single path SC decoder, that is, a metric that indicates where the possibility of the elimination of the correct path is relatively higher than other bit coordinates. When the difference between $P({\hat{u}}_{i}\left[l\right]=0|{\hat{u}}_{0}^{i-1}\left[l\right],y_{0}^{N-1})$ and $P({\hat{u}}_{i}\left[l\right]=1|{\hat{u}}_{0}^{i-1}\left[l\right],y_{0}^{N-1})$ is small for every l∈[1,L] due to small $|\lambda_{0}^{i}\left[l\right]|$ (c.f. (6)), the possibility of bit error at *i* for every l∈[1,L] is relatively high. Given that we are not aware of the location of the correct path among the *L* paths before path splitting at each decoding step *i*, this could be the coordinate that the correct path can be eliminated. To indicate these coordinates, we use the probability ratio

(9) $$\Delta_{i}=\frac{P({\hat{u}}_{0}^{i}\left[2L\right]|y_{0}^{N-1})}{P({\hat{u}}_{0}^{i}\left[1\right]|y_{0}^{N-1})}$$

Clearly, $P\left({\hat{u}}_{0}^{i}\left[2L\right]\right)<P\left({\hat{u}}_{0}^{i}\left[1\right]\right)$. The large value of this ratio indicates that $P({\hat{u}}_{0}^{i}\left[2L\right])$ and $P({\hat{u}}_{0}^{i}\left[1\right])$ are close. This implies that the correct path could be among *L* worst paths in terms of path metric due to high possibility of bit error for all the paths.

In practice, we prefer addition over division. Hence, we take the logarithm of (9) and as $P\left({\hat{u}}_{0}^{i}\left[2L\right]|y_{0}^{N-1}\right)<P\left({\hat{u}}_{0}^{i}\left[1\right]|y_{0}^{N-1}\right)$, we multiply it by -1 to have a positive metric.

(10) $$\begin{array}{c} \delta_{i}=-\log\left(\Delta_{i}\right)=-\log\left(\frac{P({\hat{u}}_{0}^{i}\left[2L\right]|y_{0}^{N-1})}{P({\hat{u}}_{0}^{i}\left[1\right]|y_{0}^{N-1})}\right)\\ =\log P\left({\hat{u}}_{0}^{i}\left[1\right]|y_{0}^{N-1}\right)-\log P\left({\hat{u}}_{0}^{i}\left[2L\right]|y_{0}^{N-1}\right)\\ =-\left(-\log P({\hat{u}}_{0}^{i}\left[1\right]|y_{0}^{N-1})\right)+\left(-\log P({\hat{u}}_{0}^{i}\left[2L\right]|y_{0}^{N-1})\right)\\ \overset{\left(4\right)}{=}PM_{2L}^{\left(i\right)}-PM_{1}^{\left(i\right)} \end{array}$$

Algorithm 1 illustrates a modified list decoder to implement the shifted pruning scheme. In this algorithm, the length of the shift is equal to κ=L/2 as assigned in line 1. In the modified SC list decoding, if the decoding fails (line 10), the shifted pruning scheme is performed on one of the bits in the 1st column of σ at every decoding attempt. The 2nd column of σ includes $\sigma_{i}$ of those bits in the first run. The maximum number of attempts equals *T*, as shown in line 12. The shifted pruning operation is performed in line 27 over only one bit at each decoding attempt. Note that in this algorithm, L can be considered a path list and a path as an object with the properties, such as the data vector, the intermediate LLR vector, partial sums vector, etc.


**Algorithm 1:** List decoder with shifted pruning.

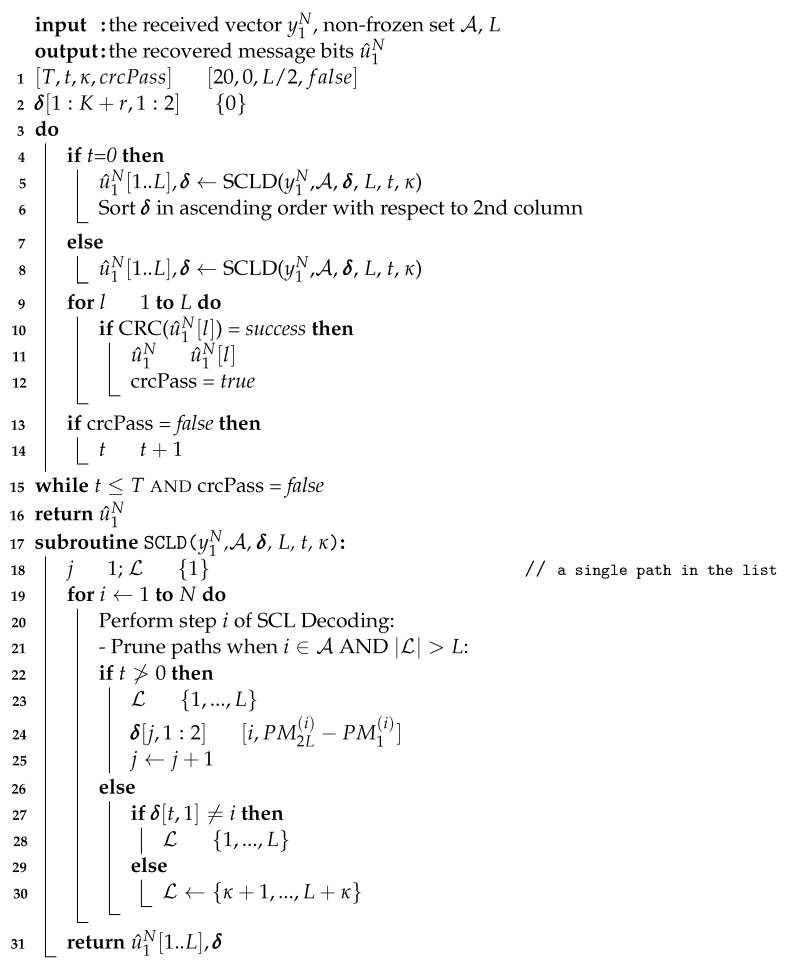




## 4. Analysis of Error Occurrence in List Decoding

**Figure 3:**
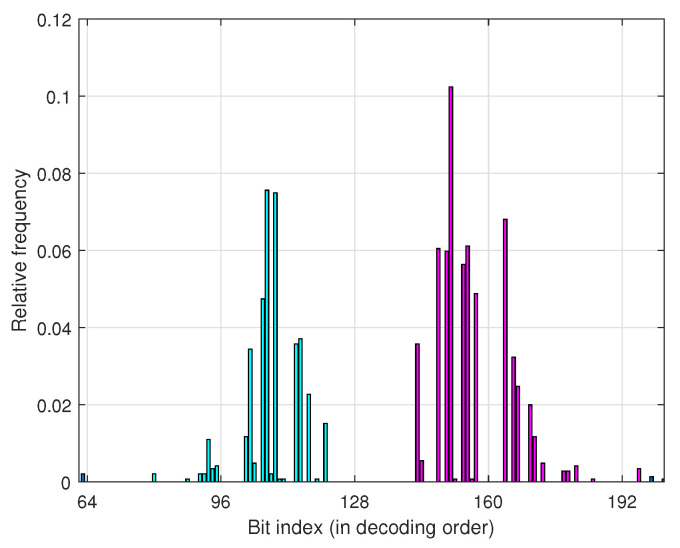
Relative frequency of elimination caused by more than one penalty over bit-channels for N=256, R=0.5 and L=8.

To understand the need for multiple shifting, in particular in the segmented scheme that is discussed later. We present a simple model for error occurrence in the list decoding in this section. Before that, let us look at some numerical results. Figure 3 shows the relative frequency of the elimination of the correct path at low-reliability bit positions. As can be seen, the relative frequencies of eliminations in the two middle 64-bit segments of N=256 are shown by different colors. Segments are an equilength ordered set of bits that are obtained by dividing a block code $x_{0}^{N-1}$ into *M* sub-blocks of size $2^{m}$ bits where $M=N/2^{m}=2^{n-m}$. One can observe a trend of increasing the frequency of elimination from the beginning of a sub-block, reaching a peak, and then decreasing the frequency. Later in this section, we will explain the reason behind this trend based on the error probability of bit channels, and then we will define a set of bits that contributes the most in the penalties.

To explain the trend observed in Figure 3, we first consider the elimination due to two penalties only, which follows the same trend. Assuming the first non-frozen bit in a segment is indexed *i*, the possibility of the second penalty occurring increases by a factor of b1 as we proceed with the decoding from bit i+b onward where b=1,2,…. In this analysis, we only consider the low-reliability bits of the segment. Note that here we are assuming the second penalty occurs on the current bit and the first penalty on one of the previous bits. For example, if the second penalty occurs on bit i+1 where j=1, the first penalty could have only occurred on bit *i*, while in case of the occurrence of the second penalty on bit i+4 where j=4, the first penalty could have occurred on bit *i*, i+1, i+2, or i+3. Now, let *j* and ν denote the index of the current bit and the number of penalties that occurred up to and including bit *j* in a segment, respectively. In general, the probability that the ν-th penalty occurs at bit j=0,1,… is

(11) $$P_{j}\left(\nu \right)\approx p_{e,j}\sum_{\begin{array}{c} B\subset \{0,\cdots ,j-1\}\\ |B|=\nu -1 \end{array}}\prod_{t}hatBp_{e,r}\prod_{s\in B^{c}}\left(1-p_{e,s}\right)$$

where $p_{e,j}$, $p_{e,r}$, and $p_{e,s}$ are the bit error probability due to channel noise for bits *j*, *r*, and *s*, respectively. The sum in (11) represents the probability of occurring ν−1 penalties out of *j* bits, which are located before the *j*-th bit. This probability follows the Poisson binomial distribution in which we consider all the combinations of ν−1 independent penalties with unequal probabilities. Although the error probability of the bit-channels is not generally independent, this model gives a good approximation. Sizing the segments properly results in the preservation of the shape of the binomial distribution shown in Figure 3. Note that there is a similarity between (13) in [7] and (11) in the sense that the probability of $E_{w}$ correcting the trajectory of SC is equivalent to the probability of |B|+1 accumulated penalty over positions B∪{j} where B⊂{0,…,j−1} assuming the correct path is still in the list. However, we here consider every B⊂{0,…,j−1} with size |B|=ν−1 when decoding position *j* to obtain a union bound, while a specific sequence of flipped positions $E_{w}$ in [7] is considered.

Note that the probability of elimination of the correct path differs from the probability of ν accumulated penalties, though they look proportional. The penalties imposed on the correct path move it within the sorted list toward l=L and beyond (i.e., elimination event). The more penalties are imposed, the larger the movement within the list will be.

Based on our findings from the study on the distribution of the location of path elimination in the model above and Figure 3, i.e., the segment-wise distribution of the location of path elimination and the impact of vicinity locations on the elimination due to multiple penalties, we propose two schemes to tackle the elimination that occurs at high channel noise levels.

## 5. Toward Nested Shifted Pruning

**Figure 4:**
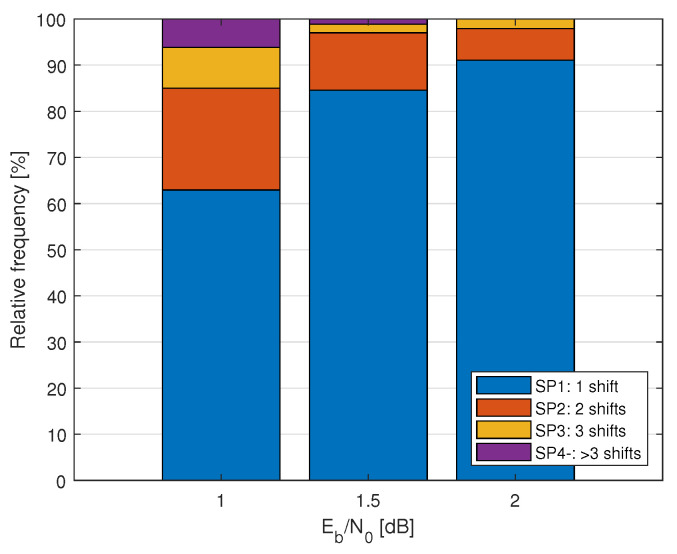
Relative frequency of the number of oracle-assisted recoveries of correct path throughout decoding by one or multiple shifting of the pruning window (SPx, x = 1, 2, …) in 30,000 codewords of P(512, 256+12) under CRC12-aided list decoding with list size L=8 and κ=L.

The elimination of the correct path may not be prevented by just a one-time shifting throughout the decoding of a codeword.Let us first look at some statistics, and then we refine our scope of the investigation and propose a scheme. Suppose that there exists an oracle in the list decoder that can avoid the elimination at any bit indices by shifting the pruning window. Figure 4 shows a relative frequency of need for one shift, compared with multiple shifts of the pruning window to recover the correct path throughout the decoding process of one codeword. As this figure shows, at high SNR regimes, the number of multiple shifting is quite small.

**Figure 5:**
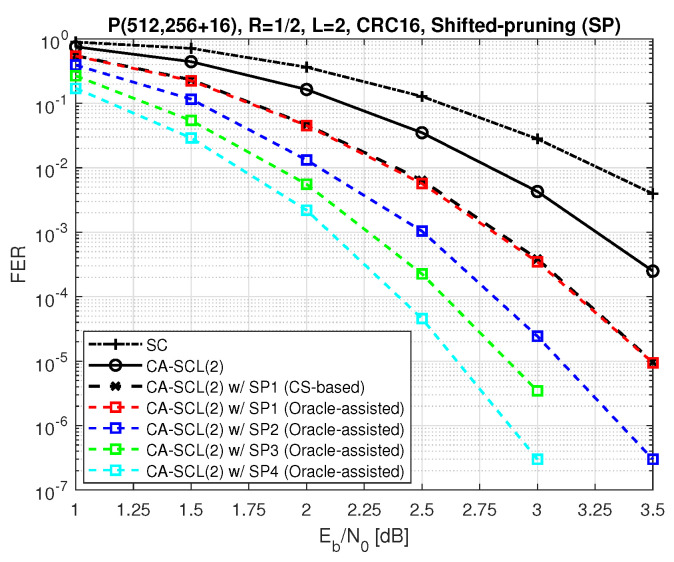
Performance of oracle−assisted list decoding with multiple shifts with κ=L.

Now, let us observe the performance improvement due to multiple shifts. Figure 5 shows that the error correction gain obtained from multiple shifts is significantly higher than one-time shifting. The notation “SP” in the figure is used for oracle-assisted shifted pruning, and x in “SPx” indicates the number of shifts applied throughout one decoding iteration to avoid the elimination of the correct path. As can be seen in Figure 5, if we use the full critical set in additional decoding attempts for the case SP1, the FER performance is expected to be as good as the oracle-assisted performance. Note that in this example, we use 16-bit CRC to significantly reduce the probability of the false detection of the correct path. As the other dashed curves show, the employment of nested shifts could improve the performance further. However, finding the right combination of the bit indices for shifting requires a massive search.

**Figure 6:**
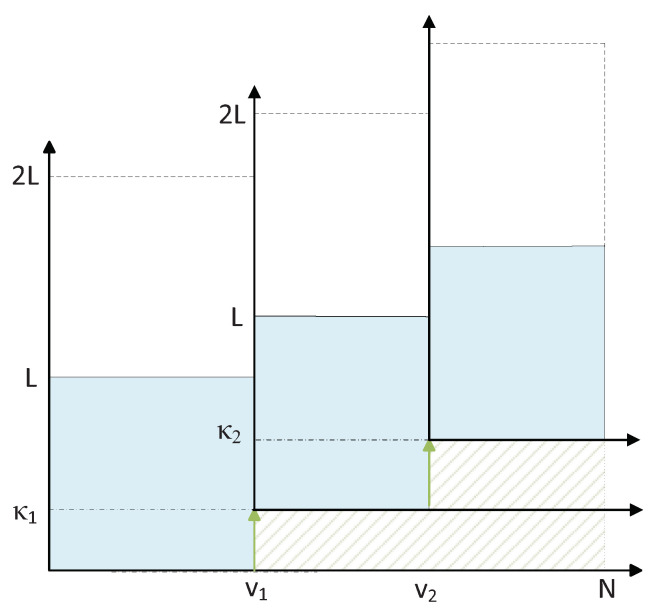
Nested shifting by *k* paths at bit $v_{1},v_{2}\in V$ during pruning operation.

Given that the error prevention by more than two shifts occurs rarely at the high SNR regimes, due to the difficulty and need for a computationally expensive search for finding the right combination of positions for more than two shifts, we only focus on double shifting in this section. Figure 6 illustrates a sketch of double shifting.

**Figure 7:**
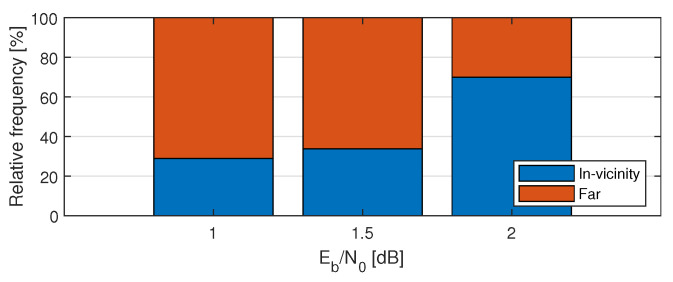
Relative frequency of the number of 2 shifts at positions in vicinity ($v_{2}-v_{1}\leq 10$) and at positions in farther distance ($v_{2}-v_{1}>10$).

Two shifts can occur in the vicinity, i.e., $v_{2}-v_{1}\leq d$ (*d* is the vicinity parameter) or far from each other in two different segments. Figure 7 shows the proportion of occurrence of these two cases at different SNR regimes.

We will cover multiple shifting occurring in different segments in Section 5.1 to reduce the complexity and the pair-shifting in the vicinity later in Section 5.2 to improve the performance at the expense of higher complexity.

### 5.1. Segmented Shifted Pruning

In segmented/partitioned list decoding [19,20], we have to use multiple short CRCs to detect the correct path in each segment. Unfortunately, this may cost us a code rate when the sum of these short CRCs is larger than a single CRC for the whole codeword, and consequently, this brings a slight performance degradation. Note that the probability of undetected error (false detection of the correct path) by a short CRC is high [21]. Considering that we need to run additional decoding in the shifted pruning scheme, the probability that an incorrect path is detected by CRC as the correct path increases significantly due to the increase in the number of iterations. Thus, the bad news is that we cannot expect to obtain the performance of nested shifts as shown in Figure 5. However, there is good news, which is that there is a significant reduction in the computational complexity by using segmented list decoding. The reduction in complexity comes from the fact that we do not need to apply the shifting on the whole codeblock. For example, in decoding P(512, 256 + 2 × 8) with two segments for which 8-bit CRCs are used, if the elimination occurs in the first segment only, we just apply the additional decoding iterations on that segment and once the correct path is recovered, the second segment may not require additional decoding attempts. Therefore, the additional computational complexity introduced by additional attempts is halved for this codeword. Note that the failures through detecting incorrect paths due to employing short CRCs are traded off by successes due to multiple shifts, one shift in each segment, and overall, the performance improvement remains almost the same at a small list size.

For computational complexity comparisons, since the block-length *N* is fixed, we drop N·logN from the computational complexity O(LNlogN), hence, we use the average list size *L* as a measure of the complexity of the shifted pruning scheme. Now, let denote the total iterations and the total decoded messages during decoding, and the number of segments by *t*, *c*, and *s*, respectively, the computational complexity of non-segmented and segmented decoding schemes are computed by $O\left(\left(\frac{t-c}{c}+1\right)\cdot L\right)=O\left(\frac{t}{c}\cdot L\right)$ and $O(\left(\frac{t-c}{s\cdot c}+1\right)\cdot L)$. Note that in the segmented decoding, *t* refers to the iteration in each segment; hence the total iterations are a sum of the iterations at all segments. Furthermore, t−c indicates the additional iterations performed for shifted pruning to correct the error.

### 5.2. Double-Shifting: Ordered-Pairs

**Figure 8:**
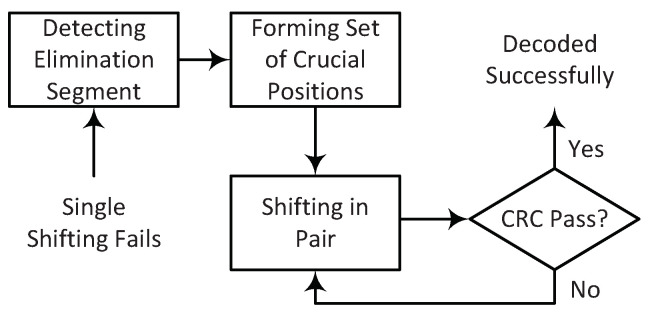
The procedure of double−shifting when the single shift at *T* positions fails.

As the numerical analysis shows, in the presence of severe noise, when the correct path is recovered by shifting the pruning window, it might be eliminated at the next consecutive low-reliability bit or a bit in the vicinity due to the bit-channels correlation. Here, we propose a scheme to avoid elimination by shifting at two close positions within a segment. Obviously, we first try to recover the correct path by a single shift. As Figure 8 shows, if the initial *T* attempts fail, multiple shifting may recover the correct path. We do not always try pair-shifting, but we perform it when the chance of recovery by pair-shifting is high. We use the following criteria: if among the top 5 out of *T* sorted positions with respect to the metric, the majority of them belong to a specific segment. This could be a sign that we are facing a low-reliability segment. Otherwise, it is not suggested to try multiple shifting, as it is quite costly in terms of time and computational complexity.

**Figure 9:**
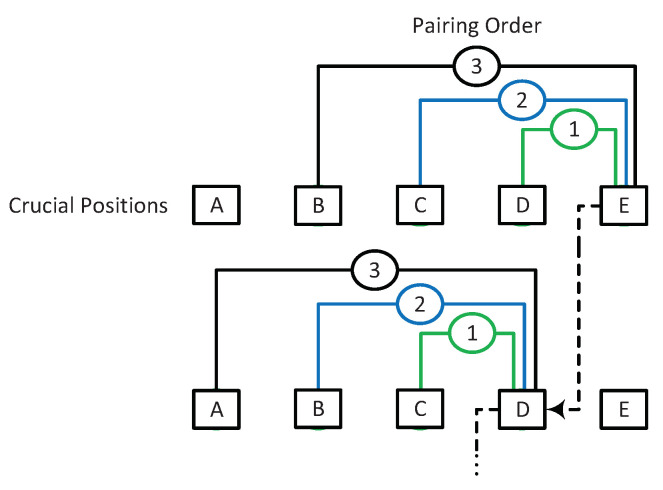
Position pairing scheme for double shifting. Positions A−E belong to one segment where A < B < C < D < E. The pairing starts from E: (E,D), (E,C), (E,B), then D: (D,C), (D,B), and so on.

After detecting a low-reliability segment, we sort the bit indices that belong to this segment, appearing among the *T* positions, in descending order. Let us call these bit indices critical bits of the segment. The pairs are selected as shown in Figure 9. That is, the first sorted position is paired with the second position. If the decoding fails, the first position is paired with the third position. After trying all the combinations of the first position with the three positions in the vicinity, we try all the combinations of the second position paired with each of the three positions before that. Let us denote the number of positions in the vicinity for pairing as π. This parameter should not be large as the possibility of elimination for the second time is higher in the close vicinity due to a stronger correlation between the bit-channels in the vicinity. It is needless to mention that a large π may not increase the chance of recovery, but it adds a significant number of failed iterations. One can choose π=2 instead of 3 in the vicinity to reduce the complexity because the second time elimination predominantly occurs at a close position. Note that the vicinity parameter *d* is different with π because in between $v_{1}$ and $v_{2}$, there might be frozen bit-channels or non-frozen reliable bit-channels.

## 6. Hardware Implementation Considerations

As the shifted pruning scheme performs in a similar fashion as the conventional list decoding, the same design can be used for implementing this scheme. The only difference is in re-running the decoder and shifting the pruning window at certain bit positions. These candidate bit-positions require a different metric. As the path metric used in this work is based on the approximate LLR-based path metric, the metric can be easily computed throughout the first run and sorted at the end. This obviously requires additional circuitry for a network sorter.

In terms of the space complexity of the segmented decoding, note that all the intermediate LLRs in the second segment are computed only based on the channel LLRs, which are the same for all the paths, and the partial sums obtained from the first segment. If we decode the first segment correctly, the partial sums of the correct path used for the second segment can be stored or can be computed simply by $u_{0}^{N/2-1}G_{N/2}$. Hence, if we choose to store the partial sums of the correct path in the first segment to be used for the second segment, we only need an additional N/2 bit memory. On another note, due to the weak protection of the short CRCs which increases the probability of false detection in the additional decoding attempts, as well as the need for storing the intermediate LLRs and partial sums, we do not recommend the use of more than two segments. As one can observe, it will add to the space complexity of the shifted pruning scheme as well.

This scheme can also be simplified based on the special nodes as was discussed in [22]. As mentioned before, the decoding of the second segment only requires the channel’s LLRs and partial sums from the first segment. Hence, this scheme is compatible with the fast and simplified version of the list decoding as well.

## 7. Numerical Results

**Figure 10:**
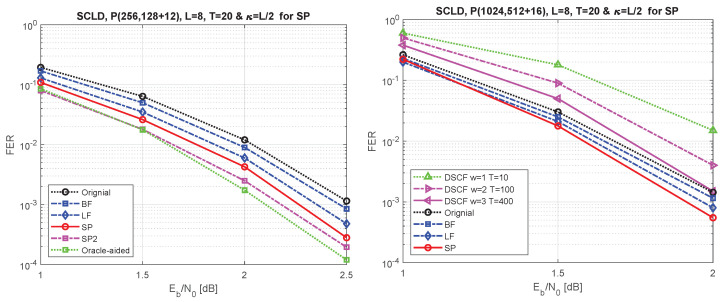
FER performance for P(256, 128+12) on the (**left**) and P(1024, 512+16) on the right under the original SC list decoding, bit-flip (BF) for list decoding [10], list-flip (LF) scheme [14], shifted-pruning (SP), and double-shifting (SP2). The (**right**) figure also compares the performance of dynamic SC flip decoding [8] with various numbers of iterations.

To evaluate the performance of the proposed metric (10), polar codes of length $N=2^{8}$ and $2^{10}$ with the code rate of R=K/N=0.5 are constructed using density evolution under Gaussian approximation [23] while optimized for high SNRs with design-SNRs of 2 dB and 3 dB, respectively. The LLR-based CRC-aided (CA) SCL decoder is used with 12-bit and 16-bit CRC generator polynomials of 0xC06 and 0x1012. Here, the coefficients of the generator polynomials are represented by hexadecimal numbers. Note that the most significant bit of a polynomial is always 1, thus, by convention, it is not shown in the hex representations. For example, 0xC06 is used to represent $g\left(x\right)=x^{12}+x^{11}+x^{10}+x^{2}+x$. Note that we use the same polynomials for the rest of the simulations as well. Figure 10(left) compares the frame error rate (FER) performance of the proposed metric with the performance of the conventional CRC-aided (CA) SCL decoding (SCLD) and [10,14]. Furthermore, this figure illustrates the FER performance gain of double-shifting under the ordered-pair scheme, SP2, in comparison with single shifting. It seems that double shifting approaches the performance of the oracle-aided with a single shift. We observed in [11] that the oracle-assisted gain can be obtained by *T*, equal to the size of the critical set, which is large and imposes a huge complexity, while double shifting can approach that performance with less complexity.

Similarly, you can observe the performance comparison for P(1024, 512+16) in Figure 10(right). Additionally, the performance comparison of the dynamic SC-flip (DSCF) decoding with w=1,2,3 and T=10,100,400, respectively, shows that the performance of DSCF decoding with w=3 and T=400 can reach the performance of the conventional list decoding without applying the shifted pruning scheme. Considering the low resources required by DSCF decoding, it could be a good option for applications that tolerate variable latency and require a small footprint.

**Figure 11:**
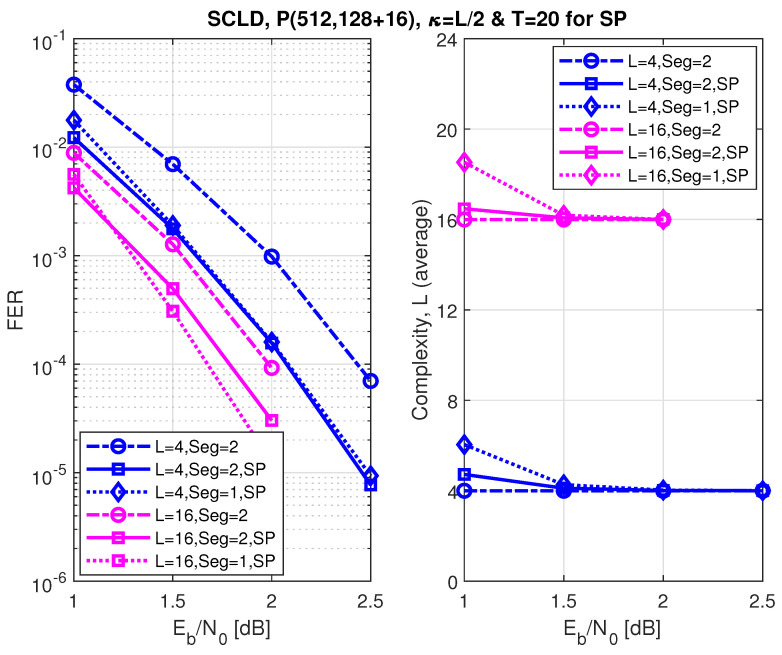
Error correction performance under segmented CA-SCL decoding with shifted-pruning (SP). Observe that SP2 is equivalent to “Seg=2, SP”.

**Figure 12:**
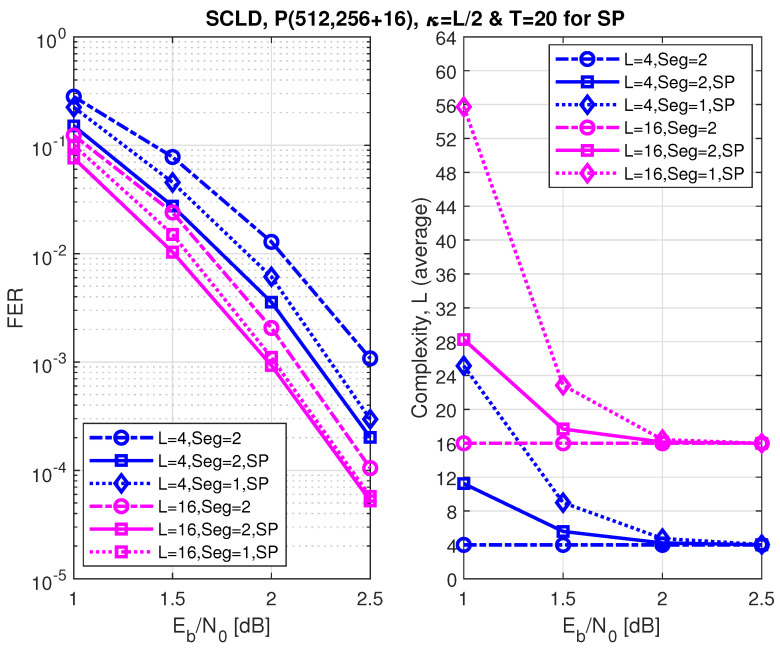
The FER performance of P(512, 256+16) under segmented CA-SCL decoding with shifted-pruning (SP). Observe that SP2 is equivalent to “Seg=2, SP”.

**Figure 13:**
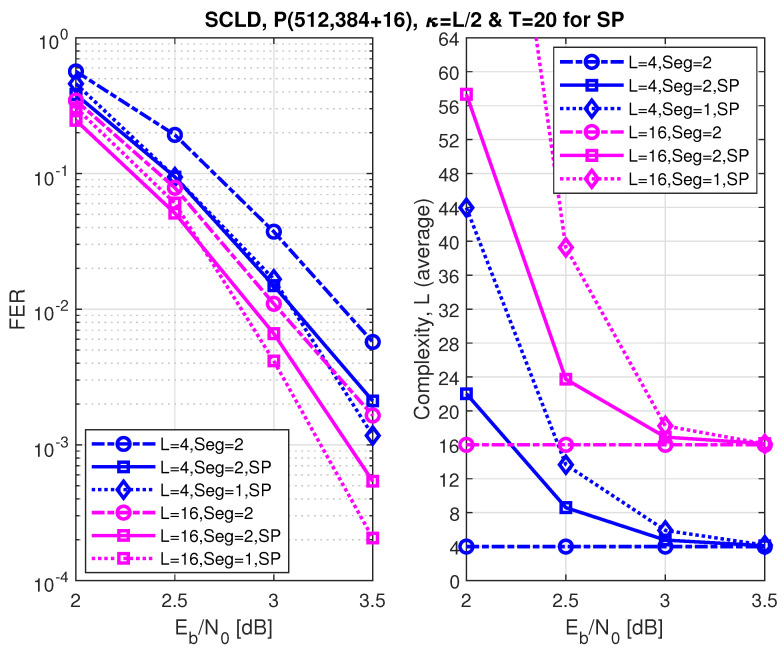
The FER performance of P(512, 384+16) under segmented CA-SCL decoding with shifted-pruning (SP). Observe that SP2 is equivalent to “Seg=2, SP”.

Figure 11, Figure 12 and Figure 13 show the FER performance and the relative complexity of the segmented/partitioned shifted pruning using one and two segments protected by 16-bit and two 8-bit CRCs with the generator polynomials 0x1021, and 0xA5, respectively. The codes P(512, 128+16), P(512, 256+16), and P(512, 384+16) were constructed by the DE/GA method, and design-SNR = 1, 2, and 2, respectively. The main significant observation is that the complexity of the segmented list decoding under shifted pruning is significantly smaller. The performance of segments and non-segments (where seg=1 in the figures) at low and high code rates shows that as the list size increases from 4 to 16, the segmented decoder underperforms compared to the non-segmented decoder. This is caused by the weak protection of the short CRCs in the segmented decoder where the probability of undetected error increases at a large list size of L=16 and T=20 (the probability of undetected error is four times of the case when L=4). Note that the relative distribution of the low-reliability bit-channels at different rates is different. While at low and high code rates, these low-reliability bit-channels are mostly located at segments 2 and 1, respectively; at the medium code rate, this distribution is different, which makes the segmented and non-segmented decoders, even in terms of additional iterations, target the segments. The improvement of the segmented decoder over the non-segmented one at a medium code rate comes from the fact that, given a limited number of iterations (T=20), the decoder will obtain more trials for each segment.

## 8. Conclusions

In this paper, we propose a new metric for shifted pruning scheme that improves the performance of the shifted pruning scheme significantly in comparison with the available metrics; also, this metric is computationally simple and works with LLR-based SCL decoding. Then, a double-shifting scheme is suggested to prevent a portion of errors that require two shifts by an oracle. This scheme improves the FER performance at a reasonable complexity cost. Finally, we adapt the segmented decoding to the shifted pruning scheme, which results in a significant reduction in the complexity of the decoding process, as the number of node visits decreases by targeting smaller segments for additional iterations.

## Data Availability

Not Applicable.
